# Flavonoids regulating NLRP3 inflammasome: a promising approach in alleviating diabetic peripheral neuropathy

**DOI:** 10.1007/s10787-025-01729-7

**Published:** 2025-04-09

**Authors:** Saumya Khanna, Sachindra Kumar, Pratyasha Sharma, Rajni Daksh, Krishnadas Nandakumar, Rekha Raghuveer Shenoy

**Affiliations:** Department of Pharmacology, Manipal College of Pharmaceutical Sciences, Manipal Academy of Higher Education, Manipal, Karnataka India 576104

**Keywords:** Diabetic peripheral neuropathy, Inflammasome, NLRP3, Flavonoids, Hyperglycemia, Drug delivery

## Abstract

A common and serious side effect of diabetes is diabetic peripheral neuropathy (DPN), which is characterised by gradual nerve damage brought on by oxidative stress, chronic inflammation, and prolonged hyperglycemia. Studies identify NLRP3 inflammasome as a key mediator in the pathogenesis of DPN, connecting neuroinflammation and neuronal damage to metabolic failure. Because of their strong anti-inflammatory and antioxidant qualities, flavonoids, a broad class of naturally occurring polyphenols, have drawn interest as potential treatments for DPN. The various ways that flavonoids affect the NLRP3 inflammasome and their potential as a treatment for DPN are examined in this review. It has been demonstrated that flavonoids prevent NLRP3 activation, which lowers the release of pro-inflammatory cytokines including IL-1β and IL-18 and causes neuroinflammation. Flavonoids work mechanistically by reducing oxidative stress, altering important signalling pathways, and blocking the activities of NF-κB and caspase-1, which are both essential for the activation of the NLRP3 inflammasome. Preclinical research has shown that flavonoids have strong neuroprotective benefits, and few clinical evidence also points to the potential of flavonoids to improve nerve function and lessen neuropathic pain in diabetic patients. The current review emphasises how flavonoids may be used as a treatment strategy to target inflammation in DPN caused by the NLRP3 inflammasome. By targeting important inflammatory pathways, flavonoids provide a new way to slow the progression of this debilitating illness. Further investigation into the mechanisms, clinical translation, and novel drug delivery techniques could enhance the therapeutic efficacy of diabetic peripheral neuropathy.

**Graphical abstract:**

**Figure:**
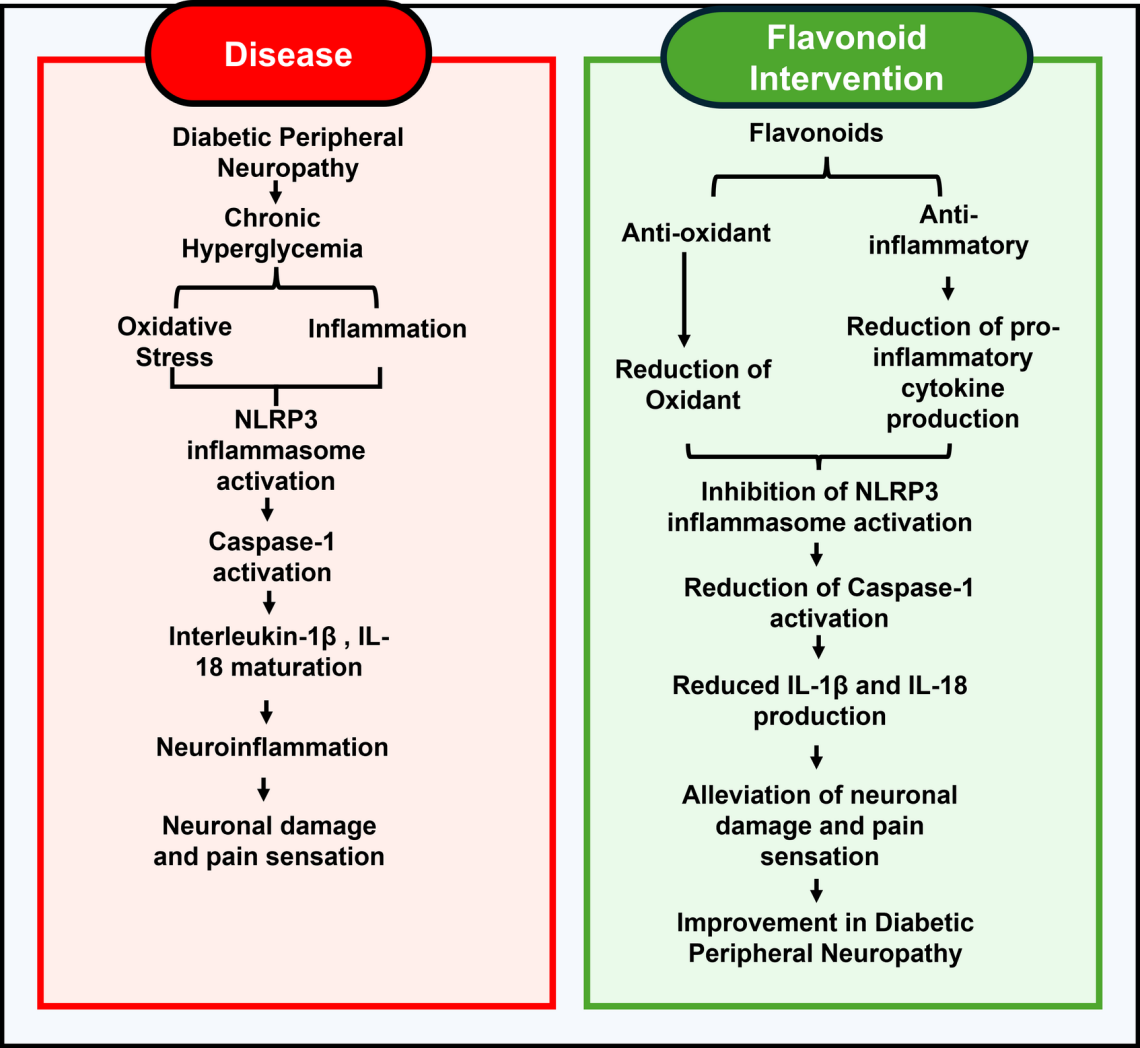
Flavonoid intervention in Diabetic Neuropathy and its role in NLRP3 Inflammasome

## Introduction

Diabetic neuropathy (DN) is one of the most frequent chronic complications of diabetes mellitus which occurs in a later stage (Chen and Song 2024) and involves both peripheral and central nerves, particularly the former, known as DPN (Diabetic Peripheral Neuropathy), which affects about one-third of patients with diabetes. Several metabolic abnormalities are the causes of elevated risk of various diabetes complications, including neuropathy in patients suffering from Type 2 Diabetes Mellitus (Oyewande et al. 2020; van der Pouw Kraan et al. 2015). This complication is characterized by pain, paraesthesia, numbness, and loss of sensation which are among the positive and negative clinical signs and symptoms that result from the progressive loss of nerve fibres (Yagihashi et al. 2007).

DPN has become a global health challenge as the number of diabetic patients increases worldwide. Diabetes has affected an estimate of 537 million adults worldwide between the ages of 20 to 79 (10.5% of all adults in this age range) (Kumar et al. 2024). The prevalence of diabetes mellitus in India is higher at 4.3%, in comparison to 1–2% in the West. Asian Indians are likely to be more susceptible to insulin resistance and cardiovascular mortality. The incidence of diabetic neuropathy (DN) from Southern India revealed that 19.1% of type 2 diabetic patients suffered from peripheral neuropathy. Around 25–50% of people with diabetic neuropathy can suffer from neuropathic pain, and the effects of this condition go well past the clinical signs and symptoms (Strand et al. 2024). Based on various meta-analysis studies across the world, the prevalence of DPN in Africa was found to be 38% in type-2 diabetic patients, 21% in Asia, mostly in India, 46.5% in Caribbean and South America, with high prevalence in Brazil and based on a population based study in the US around 14% younger adults and 42% older adults were suffering from DPN (Savelieff et al. 2025).

Currently, many drugs have been used as a treatment for Diabetic Neuropathy, like Gabapentin, Pregabalin, TCA (Tricyclic Antidepressants), and SNRIs (Serotonin and norepinephrine reuptake inhibitors). Second-line treatment drugs include capsaicin patches, opioids, and lidocaine patches, and third-line treatment includes strong opiates. Still, all these drugs come with a lot of limitations, like drowsiness, addiction, limited efficacy, and serious adverse effects (Cohen et al. 2015; Khdour 2020). Despite advances in the therapy of diabetes complications including DPN, still, there is a deficiency of effective therapeutic agents (Kabir et al. 2021).

Flavonoids are a broad group of secondary metabolites, recognized to possess a diverse range of biological activities, including antioxidant, anti-inflammatory, anti-diabetic and diabetes-related complications like diabetic neuropathy, anti-microbial, anti-cancer, neuroprotective, and cardioprotective-properties (Panche et al. 2016; Testa et al. 2016). In addition, inflammation is a critical aspect of Diabetic Neuropathy and flavonoids are known to act by inhibiting the inflammation process. Inflammasomes are the groups of multiple proteins found in immune cells initiating inflammatory reactions (Demir 2020; Sartor et al. 2002). Among various inflammasomes NLRP3 (nucleotide-binding domain, leucine-rich-containing, pyrin domain-containing-3) inflammasome is known to play a critical role in triggering an immune response thereby causing nerve damage in diabetic neuropathy (Li et al. 2022a, b; Sun et al. 2019).

This review highlights the potential effects of flavonoids on NLRP3 inflammasome and various mechanisms involved in diabetic neuropathy and discuss about the current preclinical and clinical research with respect to therapeutic effect of flavonoids in diabetic neuropathy and various novel techniques to tackle the problems associated with the bioavailability of flavonoids along with their advantages and limitations. Therefore, in this review, we systematically summarize the molecular mechanisms of flavonoids in regulating NLRP3 inflammasomes and their major role in diabetic neuropathy. This could be helpful to promote natural sources as potential therapeutic options designed to ameliorate neuropathic pain or functional changes associated with diabetic neurons.

## Flavonoids: types and sources

Flavonoids are a group of plant-derived compounds with numerous potential health benefits, including antioxidant, anti-inflammatory, anti-cancer, anti-diabetic, neuroprotective, and cardioprotective effects (Tatipamula and Kukavica 2021). Over 9000 flavonoids have been found and identified in a variety of plants. These substances show a variety of biological effects and have gained a lot of interest in pharmacology, medicine, and nutrition because of potential health advantages (Gao et al. 1999). The various types of flavonoids along with their sources and chemical structures are shown in the Table 1.

**Table 1:**
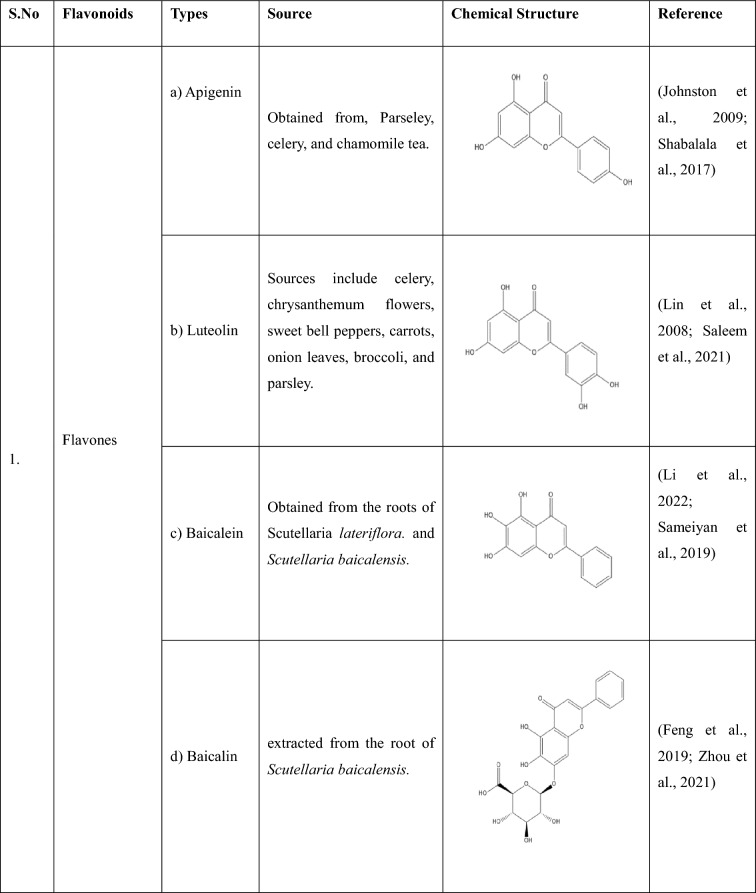

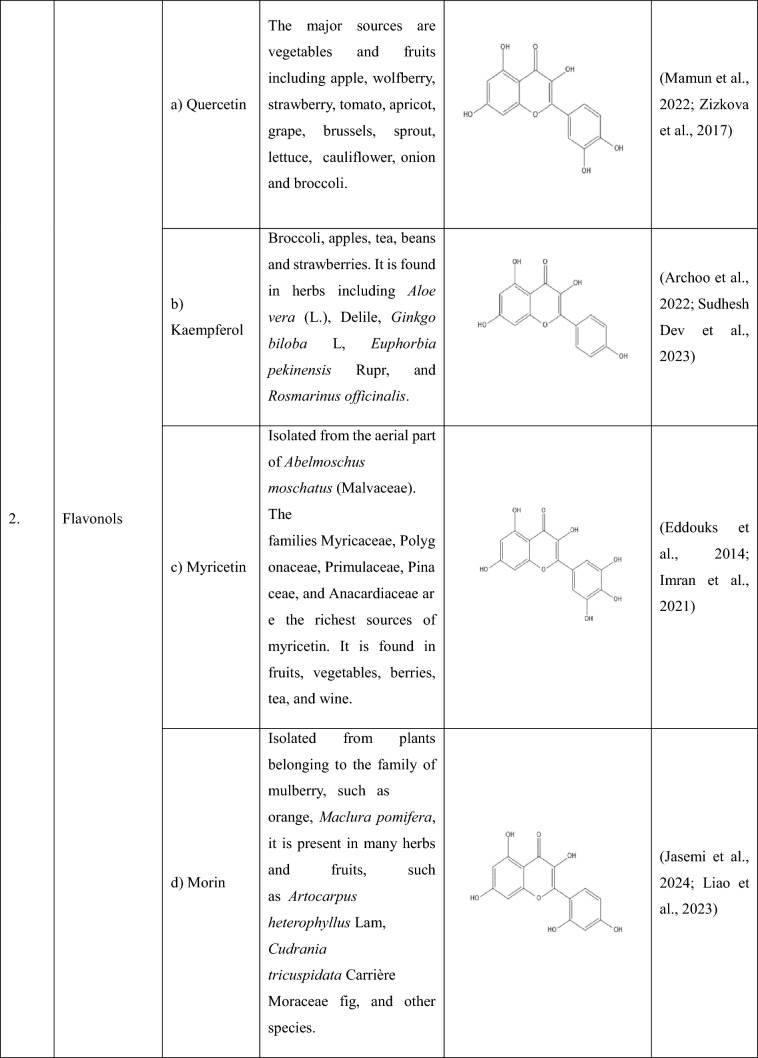

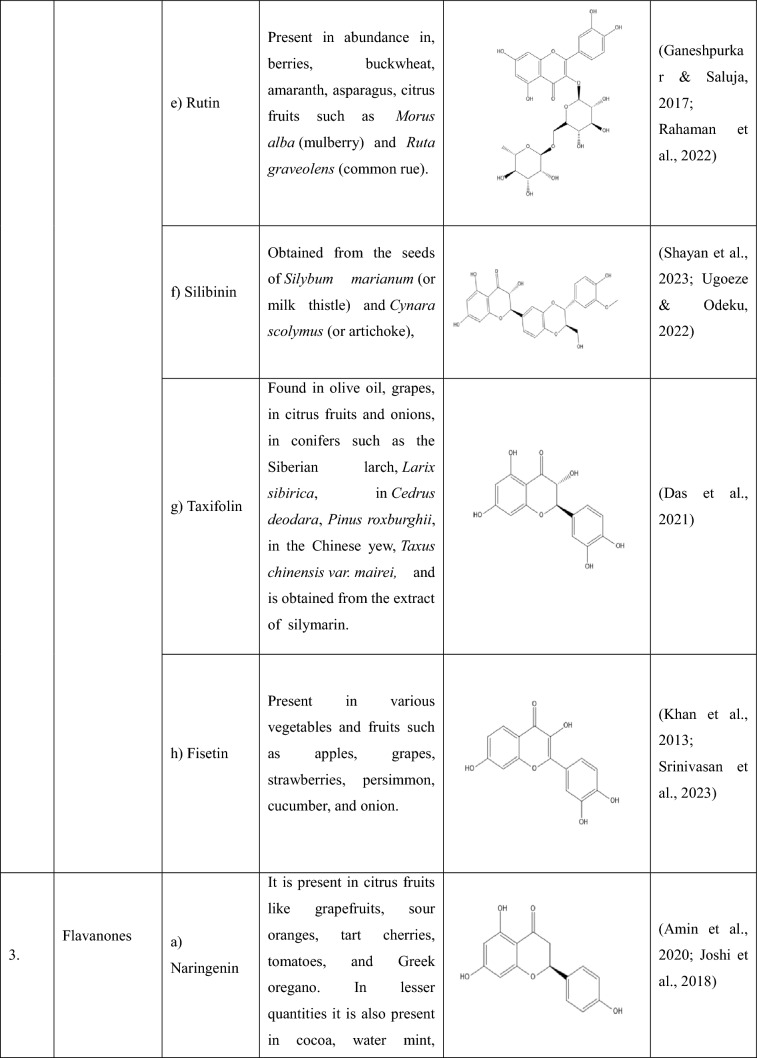

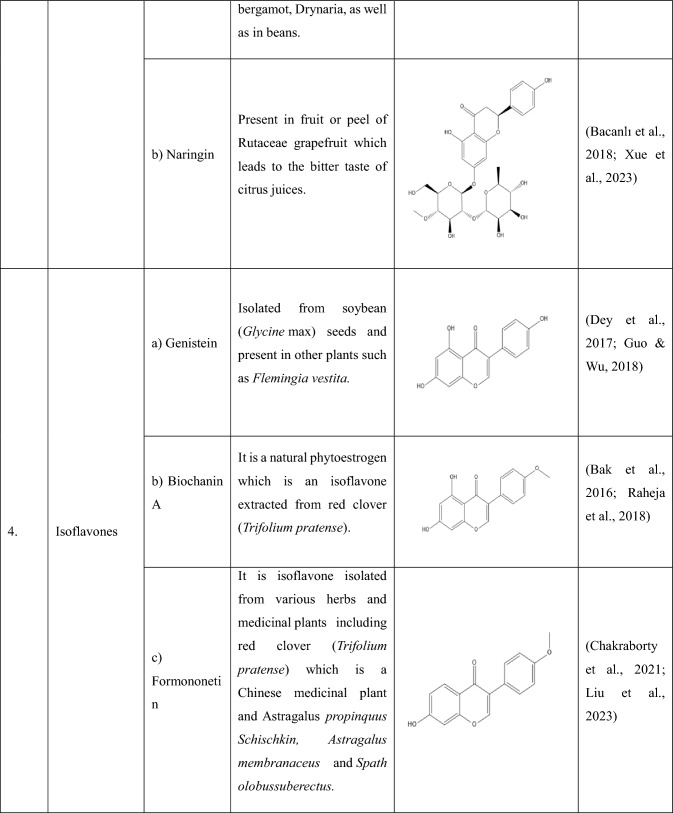

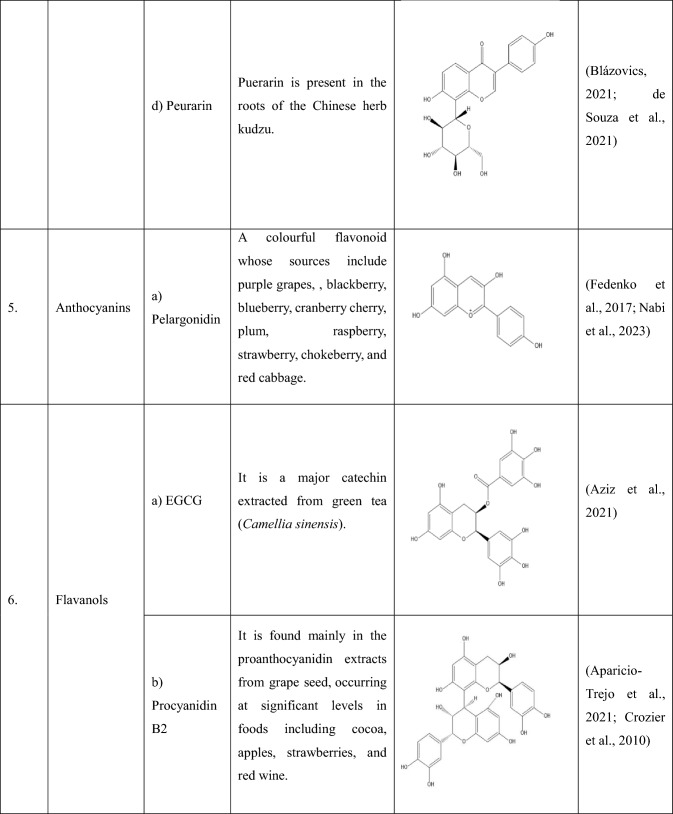

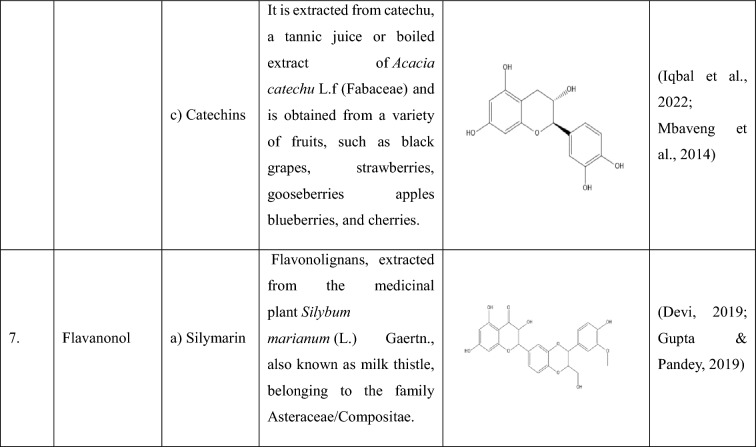
The following table provides the list of flavonoids and their types, along with their sources and chemical structure

### Pharmacokinetic properties of flavonoids

**Fig. 1:**
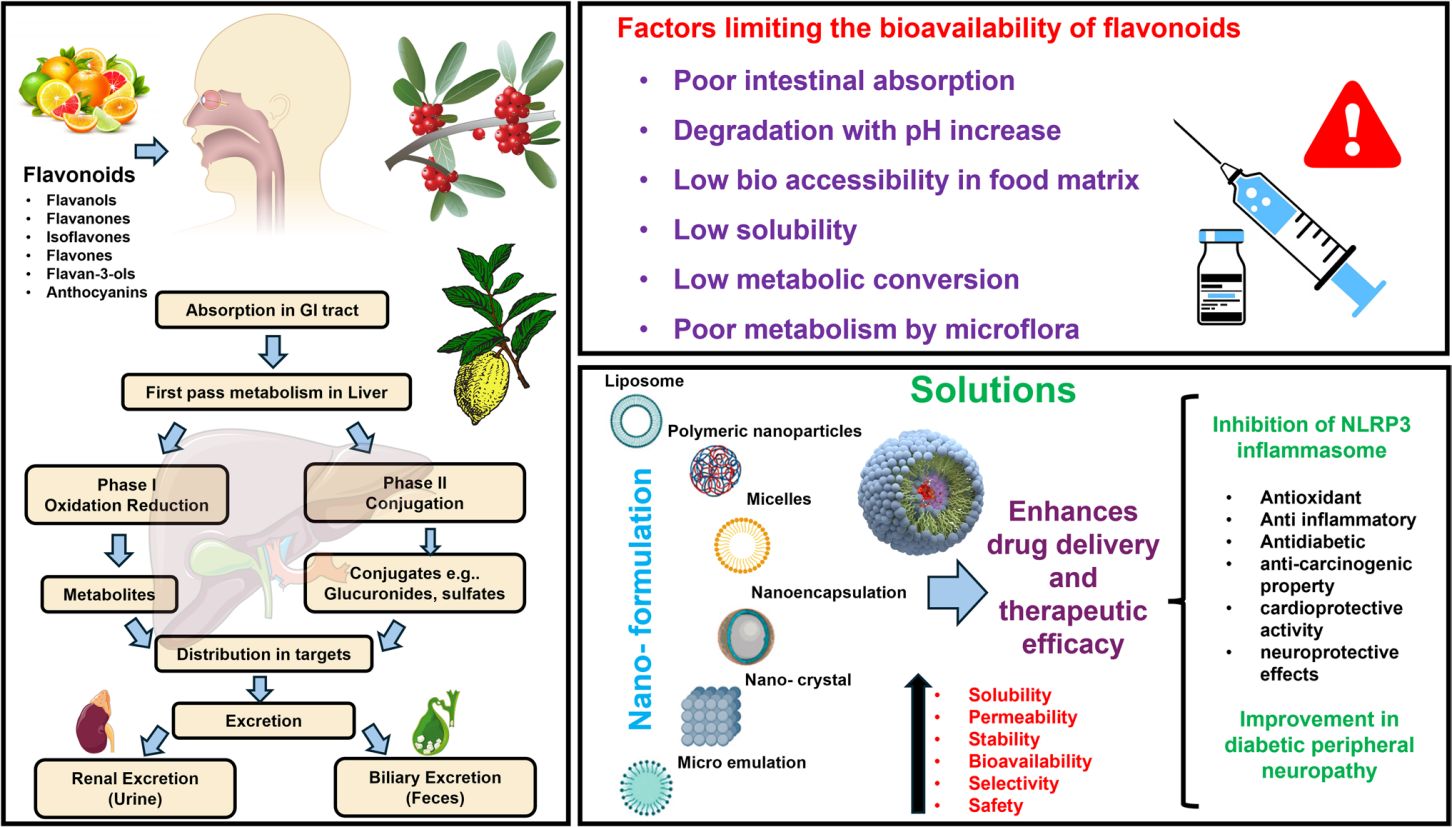
Pharmacokinetic properties of flavonoids and factors affecting the bioavailability and approaches to improve the therapeutic efficacy. The flavonoids are converted to glycosides in the small intestine by metabolic processes after ingestion. The bacterium in the colon transforms a significant portion of the flavonoids that are consumed into phenolic acids. After first pass metabolism and get further metabolized in the liver via phase I and phase II metabolism followed by excretion via kidneys or reach the target tissues. Various factors, including low solubility, low metabolism in the liver, colon, and small intestine, degradation with increase in pH and increased rate of degradation, reduces the bioavailability of flavonoids.

In nature, flavonoids appear in the form of glycosides and aglycones (free form) and are also present in the form of methylated and acetylated derivatives (Cassidy and Minihane 2017). These flavonoids are obtained from various sources such as foods and beverages among which, fruits, vegetables, flowers and seeds contain the highest content of flavonoids (Dias et al. 2021). Absorption of the flavonoids take place in the small intestine, where the lactase phlorizin hydrolase hydrolyses flavonoid glycosides into respective aglycones (Cassidy and Minihane 2017; Hollman 2004). Most of the flavonoid glucosides need to be deglycosylated in the small intestine prior to absorption; however, the structure and location of the sugar substitution determine the rate at which this process occurs. Enzymes hydrolyse the flavonoids that are not deglycosylated in the colon, helping them to be reabsorbed or excreted in feces (Dias et al. 2021). After the absorption of flavonoids, they undergo the phase I and phase II metabolism, which includes the process of sulfation, glucuronidation, methylation, and glycine conjugation (Sandoval et al. 2020). Phase I involves hydroxylation of the aromatic rings after which, the phase II metabolism occurs by conjugation of enzymes, glutathione S-transferase, UDP (Uridine 5′-diphospho)-glucuronosyltransferase and catechol *O*-methyltransferase which help in increasing the water solubility of the molecule to be excreted by the kidneys and rest of the metabolites reach the targeted tissues (Vazhappilly et al. 2021). The pharmacokinetic properties of flavonoids are illustrated in Fig. 1.

## Pathophysiology of diabetic peripheral neuropathy

The pathophysiology and pathogenesis of diabetic neuropathy is multifaceted, involving several interrelated mechanisms, including Hyperglycemia, Insulin resistance, Dyslipidaemia, and Oxidative stress (Eid et al. 2023; Feldman et al. 2019; Pang et al. 2020; Yagihashi et al. 2007). Insulin signalling is essential for sustaining neuronal glucose absorption and energy metabolism, and its resistance interferes with important processes necessary for the health and function of neurons, contributing to the blooming of diabetic neuropathy, especially in those with type 2 diabetes (Grote et al. 2011). Insulin resistance speeds up the development of neuropathy, contributing to neural dysfunction, and the impairment of neurotrophic support intensifies neuronal deterioration (Grote and Wright 2016).

Excessive amount of glucose in circulation because of insulin resistance leads to hyperglycemia, which plays a crucial role in the development of diabetic neuropathy through multiple biochemical pathways. In the polyol pathway, aldose reductase reduces excess glucose to sorbitol, which is subsequently changed into fructose by sorbitol dehydrogenase (Thakur et al. 2021). The consequent build-up of fructose and sorbitol in nerve cells results in cellular damage and osmotic stress. The non-enzymatic glycation of lipids, proteins, and nucleic acids is another way hyperglycemia encourages the production of advanced glycation end-products (AGEs). Inflammatory reactions and oxidative stress are brought on by AGEs binding to their receptors (RAGEs), which harm neurons (Lukic et al. 2008). The protein kinase C (PKC) pathway is also activated by hyperglycemia, which reduces neuronal perfusion, increases vascular permeability, hinders blood flow (30), and activates NF-κB, thus disrupting DNA and contributing to neuropathy (Kaur et al. 2023).

Insulin resistance and hyperglycemia cause an increase in dyslipidaemia and oxidative damage to mitochondrial DNA, lipids, and proteins by increasing the generation of reactive oxygen species (ROS) in the mitochondria (Pop-Busui et al. 2006) and nitrosative stress, which worsens neuronal damage by raising nitric oxide (NO) levels and its reactive derivatives, including peroxynitrite (Marrazzo* et al. 2014) and thus contributing to neuropathy. Increased concentrations of free fatty acids and their byproducts build up in peripheral nerves, causing lipotoxicity, which damages cell membrane integrity and causes neuronal cells to undergo apoptosis. Damage from lipids jeopardizes neurons’ structural and functional integrity. Furthermore, dyslipidaemia triggers the production of pro-inflammatory cytokines such as TNF-α, IL-1β, and IL-6 via activating inflammatory pathways (Cai et al. 2021; Stino et al. 2020). Furthermore, it triggers inflammatory chemokine and cytokine infiltration, leading to nerve damage and inflammation (Baum et al. 2021). The ensuing chronic inflammation harms Schwann cells and neurons, which accelerates the development of neuropathy (Cheng et al. 2024).

Additionally, the metabolic abnormalities linked to diabetes have a negative impact on Schwann cells, which are crucial for myelination and supporting neurons. This malfunction accelerates the development of diabetic neuropathy by causing demyelination and a resulting decrease in nerve conduction velocity (Sima and Zhang 2014). The intricate interaction of insulin resistance, oxidative stress, hyperglycemia, and dyslipidaemia results in a toxic environment for peripheral nerves, altering the function and structure of neurons and Schwann cells which ultimately leads to diabetic neuropathy.

## NLRP3 inflammasome: a therapeutic target

An essential part of the innate immune system is the NLRP3 inflammasome, acting as a sensor for various stress signals and playing a pivotal role in inflammation (Chen et al. 2023). The NLRP3 protein, the adaptor protein ASC (apoptosis-associated speck-like protein) and pro-caspase-1, usually make up this multiprotein complex. When this complex is activated, pro-caspase-1 is cleaved into its active form, caspase-1, which then converts pro-inflammatory cytokines IL-18 and IL-1β into their active forms, causing their release and promoting an inflammatory response (He et al. 2016; Kelley et al. 2019). Multiple processes are involved in the development and activation of the NLRP3 inflammasome in diabetic neuropathy including the Priming and the Activation step (Hamilton et al. 2022). Priming step, the initial phase, entails the induction of pro-IL-1β and NLRP3. This is usually accomplished via toll-like receptors (TLRs) that recognizes damage-associated molecular patterns (DAMPs) or pathogen-associated molecular patterns (PAMPs), and triggers the NF-κB pathway (McKee and Coll 2020). In diabetic neuropathy, chronic hyperglycemia leads to peripheral nerve injury, altered nerve Fiber Na^+^, K^+^-ATPase activity, reduced nerve conduction velocity and the release of ROS which leads to the activation of NLRP3 inflammasome (Nițulescu et al. 2023). The second step involves the activation of the inflammasome, which is triggered by various stimuli, such as oxidative stress, mitochondrial dysfunction, ion flux, extracellular ATP, Lipopolysaccharide (LPS) (Y. Chen et al. 2023). There have been various studies performed which have shown that NLRP3 inflammasome has been activated by high glucose levels due to metabolic disturbance causing the activation of procaspase-1 and IL-1β (Haneklaus and O’Neill 2015) and that high glucose levels have caused increased levels of reactive oxidant species causing the activation of NLRP3 inflammasome via TRX protein (thioredoxin-interacting protein) causing the release of proinflammatory cytokines leading to neuropathic pain (Zheng et al. 2020).

Diabetic Neuropathy is also linked with the process of pyroptosis which requires the assembly of the inflammasome, the activation of inflammasome causes accumulation of GSDMD (Gasdermin D) caspase-1 cleaves it to produce the C- and N-gasdermin domains. These domains promote an inflammatory response by secreting mature IL-1β and IL-18 and due to the aggregation of N-gasdermin on cell membranes pyroptosis is caused and excessive pyroptosis can lead to chronic inflammation (Lu et al. 2020; Xu et al. 2021). A study found that Jinmaitong, which is a traditional Chinese medicine helped in alleviating DPN by inhibiting the expressions of NLRP3 inflammasome and inhibited pyroptosis (Sun et al. 2021).

Some studies have also shown that, by controlling the AMPK-NLRP3 inflammasome axis, for example, the bioactive substance salidroside has been shown to reduce diabetic neuropathic pain. Salidroside prevents neuronal injury by lowering the inflammatory response through AMPK (adenosine 5’- monophosphate-activated protein kinase) activation (Zheng et al. 2021). In addition, increased inflammation and pain have been linked to the activation of the NLRP3 inflammasome, which is facilitated by TET2 (Tet methyl-cytosine dioxygenase 2) overexpression in dorsal root ganglion neurons. By modifying inflammasome activity and lowering inflammation, targeting TET2 and its regulatory pathways may open up new treatment options for diabetic neuropathic pain (Chen et al. 2022a, b).

Moreover, the therapeutic promise of targeting the NLRP3 inflammasome also extends to oncology; studies show that, depending on the tumour environment, altering this pathway can either promote or hinder the proliferation of cancer cells (Missiroli et al. 2021). NLRP3 activation may improve anti-tumour immunity in malignancies with low levels of IL-1β production, while NLRP3 inhibition may help stop tumour growth in tumours with high amounts of IL-1β (Sharma and Kanneganti 2021). Additionally, the NLRP3 inflammasome has been implicated in drug-induced organ toxicities, including hepatotoxicity and nephrotoxicity, highlighting its role in adverse drug reactions (Guan et al. 2022). Thus, targeting the NLRP3 inflammasome presents a multifaceted approach to therapy that could address not only chronic inflammatory diseases but also cancer and drug toxicity (Ozaki et al. 2015). Ongoing research is focused on elucidating the precise mechanisms governing NLRP3 activation and inhibition while exploring novel small molecules and biologics that can selectively modulate this pathway for therapeutic benefit. As our understanding deepens regarding the structural mechanisms underlying NLRP3 function and its interactions with other cellular pathways, it is likely that innovative strategies will emerge to harness its potential for treating a wide array of diseases characterized by inflammation and immune dysregulation.

## Therapeutic effects of flavonoids

**Fig. 2:**
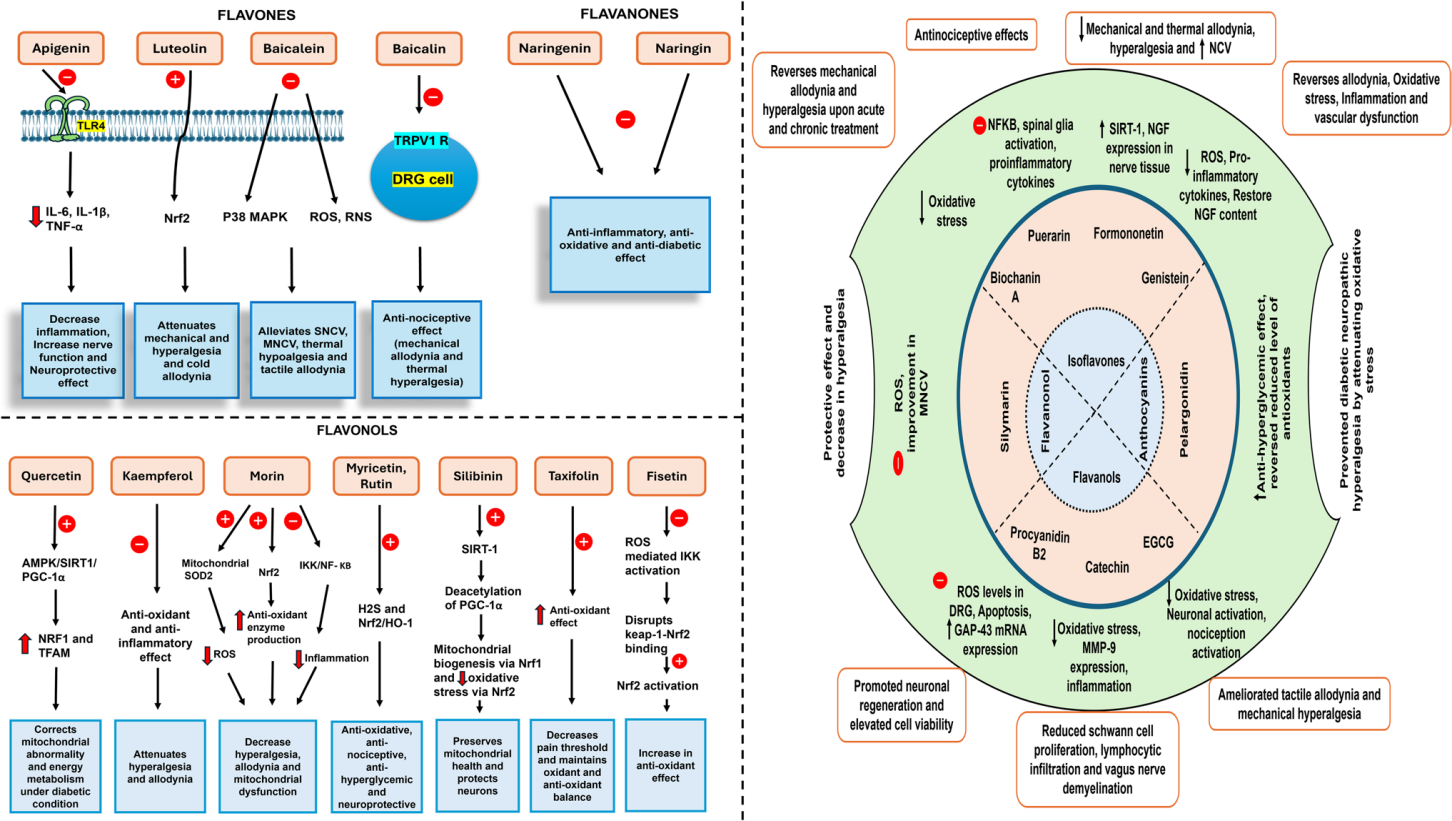
Flavonoids and their mechanism of action in Diabetic Neuropathy. In diabetic neuropathy, the picture illustrates how the various flavonoid subclasses—flavones, flavanones, flavonols, isoflavones, flavanonols, and anthocyanins—work. It draws attention to how they might lessen oxidative stress, inhibit pro-inflammatory cytokines, enhance nerve function, and lessen neuronal damage. Under each category, particular flavonoids help improve neuroprotection in diabetes and reduce neuropathic pain.

**Fig. 3:**
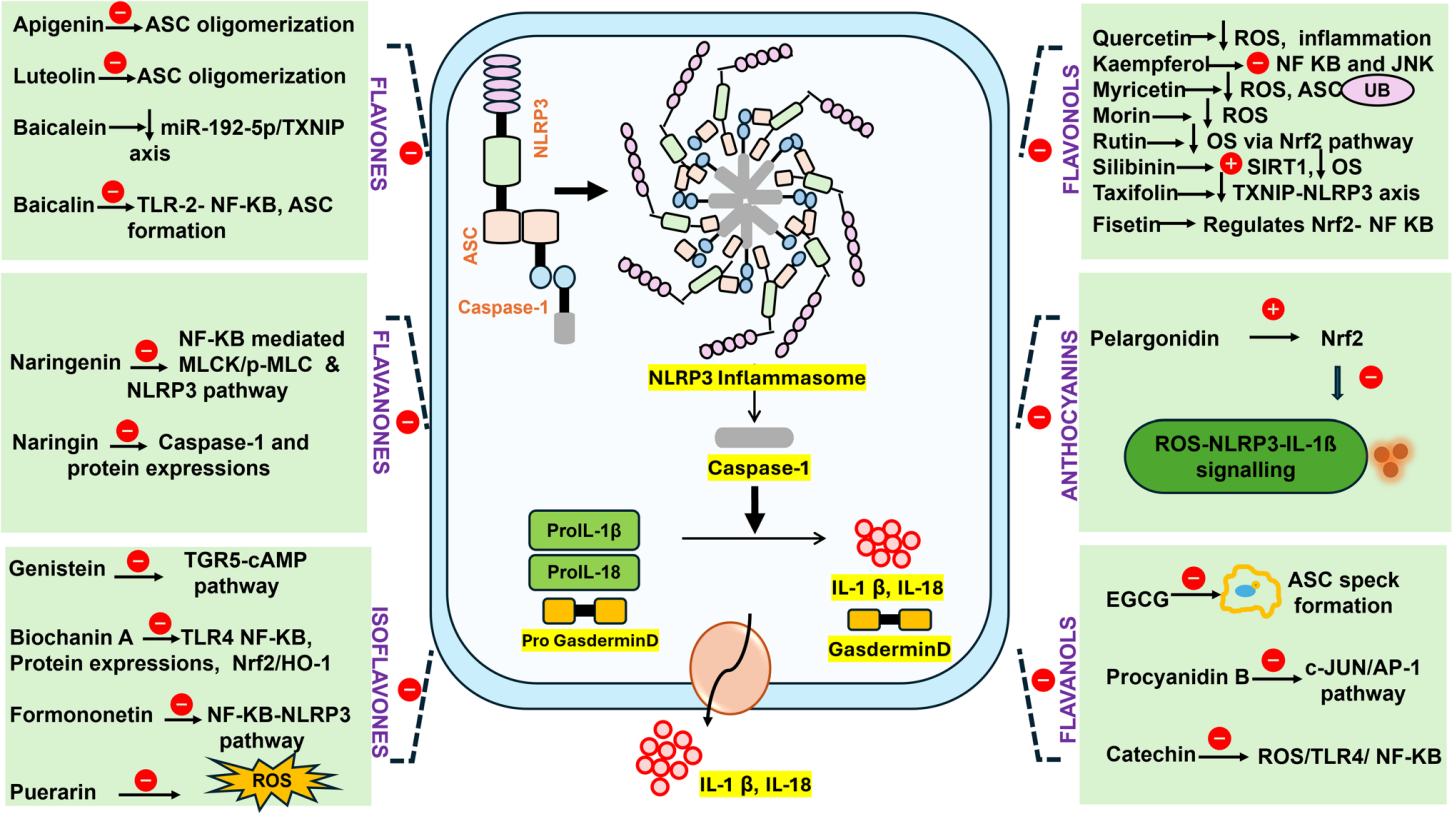
Mechanism of action of Flavonoids in Diabetic Neuropathy. The illustration shows how flavonoids affect NLRP3 components in diabetic neuropathy. Inflammasome priming, ASC oligomerisation, NLRP3 assembly, caspase-1 activation, ROS generation, and the release of pro-inflammatory cytokines IL-1β and IL-18 are all inhibited by flavonoids. These activities reduce neuronal damage and inflammation.

These plant-derived polyphenolic compounds have potent antioxidant properties and anti-inflammatory, and the mechanism involved in providing the benefit of flavonoids includes. Important inflammatory enzymes that flavonoids have been shown to inhibit include cyclooxygenase (COX), lipoxygenase (LOX), and inducible nitric oxide synthase (iNOS). Flavonoids can effectively reduce inflammation by altering the production of pro-inflammatory mediators such nitric oxide, leukotrienes, and prostaglandins (Maleki et al. 2019; Santangelo et al. 2007). Flavonoids can also interfere with the activation of transcription factors, such as nuclear factor-kappa B (NF-κB) and activator protein-1 (AP-1) which regulate the expression of various pro-inflammatory genes (Yoon and Baek 2005). By inhibiting the translocation of these transcription factors to the nucleus, flavonoids can suppress the production of chemokines, inflammatory cytokines, and adhesion molecules. Flavonoids possess potent antioxidant properties, which contribute to their anti-inflammatory effects. They can directly scavenge reactive oxygen species (ROS) and reactive nitrogen species (RNS), thereby reducing oxidative stress and the subsequent inflammatory response (Procházková et al. 2011; Soobrattee et al. 2005). Additionally, flavonoids can increase the expression of antioxidant enzymes, such as glutathione peroxidase, superoxide dismutase (SOD), and catalase, further enhancing the body’s defence against oxidative damage. Flavonoids have been shown to regulate the activity of various inflammatory cell types, including macrophages, lymphocytes and neutrophils. They can inhibit the release and production of pro-inflammatory mediators from these cells, as well as suppress their migration and infiltration to the site of inflammation (Comalada et al. 2005). Flavonoids can also influence the expression of genes involved in the inflammatory response. They can downregulate the expression of pro-inflammatory genes, such as those encoding cytokines (e.g., IL-6, IL-1β, TNF-α),(Tunon et al. 2009) chemokines, and adhesion molecules while upregulating the expression of anti-inflammatory genes (e.g., IL-10, heme oxygenase-1) which is shown in Fig. 2. Additionally, flavonoids have also been studied as NLRP3 inflammasome inhibitors and are important mediators of chronic inflammation. The NLRP3 inflammasome is the target of flavonoids, which may lessen the activation of pro-inflammatory cytokines and lessen the inflammatory cascade linked to neuronal injury and the mechanism of action of flavonoids on NLRP3 Inflammasome has been shown in Fig. 3. The approaches by which the flavonoids particularly work to achieve these effects are listed below, emphasizing their diverse function in preventing diabetic neuropathy and associated inflammatory processes.

1. Flavones
  (a) Apigenin- Apigenin, a (4′,5,7-trihydroxyflavone) is found in various plants, plays a significant role in diabetes-induced neuropathy, and has various antioxidant, anti-inflammatory, and anti-diabetic properties (Johnston et al. 2009). In a study, apigenin ameliorated diabetic neuropathy in rats by modulating the TLR4/MyD88 signalling pathway, which showed inhibited elevated plasma glucose levels, TNF-α, ILs, TLR4, and MyD88 expressions, thus ameliorating STZ (Streptozotocin) -induced allodynia and hyperalgesia (Fang et al. 2023; Yu et al. 2023). By preventing caspase-1 activation and disrupting the NLRP3 inflammasome assembly, apigenin reduced the amount of IL-1β produced in response to LPS (Zhang et al. 2014).
  (b) Luteolin- Luteolin, a (3′,4′,5,7-tetrahydroxyflavone) is found in various dietary sources and medicinal plants (Lin et al. 2008), and its antioxidant and neuroprotective effects has made it a potential option for treating diabetic neuropathy. Luteolin helped improve the impaired nerve functions in diabetic neuropathy by acting on the Nrf2-dependent anti-oxidant pathway (Li et al. 2015). A study showed that luteolin could inhibit NLRP3 inflammasome activation via blocking ASC oligomerization, showing that luteolin acts on the inflammasome (‘Luteolin Inhibits NLRP3 Inflammasome Activation via Blocking ASC Oligomerization’, 2021).
  (c) Baicalein- Baicalein, a (5,6,7-trihydroxyflavone) extracted from the root of *Scutellaria baicalensis*, is used as a dietary supplement, which helps in reducing pain, inflammation*,* and oxidative stress (Li et al. 2022a, b). Without reducing the rate at which diabetes causes the loss of intraepidermal nerve fibres and encouraging their regeneration, baicalein reduces MNCV (Motor Nerve Conduction Velocity) and SNCV (Sensory Nerve Conduction Velocity) deficits, thermal hypoalgesia, and tactile allodynia symptoms in DPN (Stavniichuk et al. 2011). Baicalein alleviates pyroptosis and inflammation by inhibiting the NLRP3/Caspase-1 pathway (Wang et al. 2021).
  (d) Baicalin- Baicalin (baicalein 7-d-β-glucuronate) extracted from the dried root of *Scutellaria baicalensis* Georgi. It has anti-inflammatory, analgesic, antioxidant, anti-allergy, and immunomodulatory effects (Feng et al. 2019). A study showed that baicalin administration suppressed the increase in the expression of TRPV1 in DRG of DNP rats which showed that baicalin plays a vital analgesic role in DNP and could serve as a potential compound in the treatment and prevention of DNP (P. Li et al. 2018). Baicalin suppressed NLRP3-inflammasome via TLR-2-NF-κB pathway (Ishfaq et al. 2021). Baicalin has also been shown to inhibit excessive NLRP3 inflammasome activation by inhibiting ASC formation and reducing the release of IL-1β and IL-18 from the cells (C.-G. Li et al. 2017).
2. Flavonols
  (a) Quercetin- Quercetin is a polyphenolic flavonoid present in plants and plant-derived foods and is known for various properties such as antioxidant, anti-inflammatory, anti-tumour, cardioprotective activity, etc. (Zizkova et al. 2017)., and has shown a promising effect in the treatment of DPN. Quercetin corrects mitochondrial abnormalities by activating the AMPK/PGC-1α Pathway, which helps alleviate diabetic peripheral neuropathy (Zhang et al. 2021a, b). According to one study, quercetin therapy reduced the formation of ROS and prevented the activation of the NLRP3 inflammasome, which resulted in a decrease in IL-1β, TNF-α, and IL-18 levels as well as in histopathology 72 h following a spinal cord injury (Jiang et al. 2016).
  (b) Kaempferol- Kaempferol (3,5,7-trihydroxy-2-(4-hydroxyphenyl)-4H-1-benzopyran-4-one) is found in many plants and has many properties, including, antimicrobial, anticancer, cardioprotective, neuroprotective antioxidant, anti-inflammatory, antidiabetic, analgesic properties, etc., (Shields 2017; Sudhesh Dev et al. 2023) and has shown its action on diabetic peripheral neuropathy and has helped in attenuating the development of allodynia and hyperalgesia in STZ model of DNP by inhibiting microglial activation and also by decreasing the inflammatory mediators and oxidative stress (Abo-Salem 2014). Kaempferol inhibited the activation of caspase-8, caspase-3, and NLRP1/NLRP3 inflammasomes, preventing the production of pro-inflammatory cytokines via inhibition of NF-κB and JNK (Jun N-terminal Kinase) pathways (C. Lin et al. 2019).
  (c) Myricetin- A phenolic compound which is isolated from the ariel parts of *Abelmoschus moschatuss* (Eddouks et al. 2014) and has antioxidant, anticancer, antimutagenic, cardioprotective, and antidiabetic activity (Kulkarni et al. 2016). It has shown a promising effect in the treatment of DPN, where it has helped in improving impaired nerve functions in experimental diabetic rats (Ma et al. 2022). Myricetin inhibits NLRP3 inflammasome activation which is accounted by the ROS scavenging activity of myricetin. Myricetin’s ability to scavenge ROS is responsible for the ubiquitination of ASC, whereas it also promotes NLRP3 ubiquitination, which is not dependent on ROS (Chen et al. 2019a, b).
  (d) Morin- Morin a, (3,5,7,2′,4′-pentahydroxyflavone) which is isolated in the form of a yellow pigment from various plants is shown to have antioxidant, anti-inflammatory, cardioprotective, neuroprotective, anti-diabetic, and has anti-microbial potential (Rajput et al. 2021). It has shown to have effect in DPN by exerting neuroprotection via attenuation of ROS induced oxidative damage and neuroinflammation (Bachewal et al. 2018). Morin significantly decreased the protein level of NLRP3, Therefore, inhibiting the activation of NLRP3 inflammasome induced by LPS (Tianzhu et al. 2014).
  (e) Rutin- Rutin, a (3,3′,4′,5,7-pentahydroxyflavone-3-rhamnoglucoside) found in various plants, has demonstrated a number of pharmacological activities, including antioxidant, cytoprotective, vasoprotective, anticarcinogenic, neuroprotective and cardioprotective activities (Ganeshpurkar and Saluja 2017). It has shown a potential effect in ameliorating diabetic neuropathy by lowering plasma glucose and decreasing oxidative stress via Nrf2 signalling pathway in rats (Tian et al. 2016). Rutin when administered showed reduction in ROS and malondialdehyde productions and suppressed NLRP3 inflammasome activation with decrease of IL-1β, IL-18, and TNF-α levels and attenuation of histopathology 72 h after spinal cord injury (Wu et al. 2016).
  (f) Silibinin- It is a flavonolignan which is separated from the seeds of *Silybum marianum*, also known as milk thistle, has various benefits including free radical scavenging, metal chelating, anti-oxidative, hepatoprotection and anti-carcinogenic property (Shayan et al. 2023). Silibinin has shown to improve mitochondrial health and alleviate the oxidative damage in experimental diabetic neuropathy and high glucose-mediated neurotoxicity via SIRT1 activation (Khan et al. 2024). Silibinin inhibited LPS-induced NF-κB activation and decreased the expression of NLRP3 inflammasome (Tian et al. 2017).
  (g) Taxifolin- A, unique bioactive flavonoid, available as a dietary component, has inhibitory activity against inflammation, microbial infection, oxidative stress, malignancies, cardiovascular disease, and liver disease (Das et al. 2021). Taxifolin has shown to prevent the reduction of alloxan-induced hyperglycemia-related paw pain threshold, and the oxidant–antioxidant balance in the sciatic nerve tissue therefore, making it useful in the treatment of hyperglycemia-associated neuropathy and neuropathic pain (Alay et al. 2022). Taxifolin has shown to suppress inflammatory responses of high-glucose-stimulated mouse microglia by inhibiting the TXNIP-NLRP3 Axis (Iwasa et al. 2023).
  (h) Fisetin- fisetin (3,3′,4′,7-tetrahydroxyflavone), is found in various vegetables and fruits and is reported to have antioxidant, anticarcinogenic, anti-inflammatory and anti-carcinogenic properties (Khan et al. 2013). Fisetin has shown to protect against oxidative stress and neuroinflammation-mediated functional, behavioural and biochemical deficits in DN via NF-κB inhibition and -positive modulation of Nrf2 (Sandireddy et al. 2016). Fisetin has shown to inhibit NLRP3 inflammasome by suppressing TLR4/MD2-mediated mitochondrial ROS production (Molagoda et al. 2021).
3. Flavanones
  (a) Naringenin- A, 5,7dihydroxy2(4hydroxyphenyl) 2,3dihydrochromen4one, is the aglycone of Naringin. It is one of the primary flavanone found in citrus fruits and has various properties, including antioxidant, anti-inflammatory, anti-diabetic, and anti-cancer, and has effects on the central nervous system and the cardiovascular system (Joshi et al. 2018). Naringenin has been shown to have a promising effect in case of diabetic neuropathy via its anti-diabetic as well as antioxidant and anti-inflammatory properties (Al-Rejaie et al. 2015). Naringenin has been shown to inhibit the NLRP3 inflammasome by attenuating the NF-κB mediated pathway (Zhong et al. 2021).
  (b) Naringin- A, citrus bioflavonoid, present mainly in citrus fruits, existing in the fruit or peel of Rutaceae grapefruit, is known to have various properties including antioxidant, anti-inflammatory, anti-cancer, anti-microbial, in many neurological diseases (Viswanatha et al. 2017; Xue et al. 2023) and has shown to have a promising effect in diabetic neuropathy. A study showed that the naringin–insulin combination not only attenuated the diabetic condition but also reversed neuropathic pain through modulation of oxidative–nitrosative stress, inflammatory cytokine release, and reduction in apoptosis in the STZ-induced diabetic rats (Kandhare et al. 2012a, b). Another study confirmed that naringin can regulate the NLRP3-caspase-1-IL-1β/IL-18 signalling pathway to affect the NLRP3 inflammasome by playing an anti-inflammatory role (Chen et al. 2018).
4. Isoflavones
  (a) Genistein- It is an isoflavone (7,4′-dihydroxy-6-methoxyisoflavone) found mainly in soya products and is shown to have anti-diabetic, anti-aging, antioxidant, anti-inflammatory, anti-cancer, and neurological conditions (Pandey et al. 2023; Sakai et al. 2022). Genistein has proven its action against diabetic neuropathy and is capable of conversing diabetes-associated conditions of OS (Oxidative stress), allodynia, and infection, upgrading NGF content and vascular disorders (Valsecchi et al. 2011; Weng et al. 2019). The NLRP3 inflammasome is modulated by genistein through cAMP, which binds to NLRP3 and facilitates its ubiquitination and destruction (Chen et al. 2019a, b).
  (b) Biochanin A- An isoflavonoid (4′-methoxy-5,7-dihydroxyisoflavone) is shown to have various properties, including anti-cancer, hepatoprotective antioxidant, anti-inflammatory activity, etc., (Y. Li et al. 2019; Suvarna et al. 2022). It has been shown that biochanin-A is more effective than mechanical hyperalgesia at reversing mechanical allodynia, which makes it a promising medication option for reducing neuropathic pain brought on by streptozotocin (STZ) (Chundi et al. 2016). Biochanin A is also shown to attenuate injury of spinal cord in rats during early phases by inhibiting oxidative stress and inflammasome activation via inhibition of the TLR4/NF-κB/NLRP3 signalling pathway (Li et al. 2024).
  (c) Formononetin- An isoflavonoid (7-hydroxy, 4′-methoxy isoflavone) extracted from plant *Dalbergia ecastophyllum* is known for its properties including antidiabetic, antiobesity, hepatoprotective, anticancer, antioxidant and anti-inflammatory activities (Machado Dutra et al. 2021; Sarfraz et al. 2021). According to a study, formononetin may improve neuropathic symptoms in type 2 diabetic rats by boosting the expression of SIRT1 (Sirtuin 1) and NGF in diabetic animals (Oza and Kulkarni 2020). Formononetin is shown to Ameliorate the inflammatory response in LPS-induced inflammatory injury by inhibiting the NF-κB/NLRP3 signalling pathway (Zhou and Zhang 2023).
  (d) Puerarin- An 8-*C*-glucoside form of daidzein, found in many herbal plants and has many properties, including cardioprotective, neuroprotective, antioxidative, anti-inflammatory, hypoglycemic, and inhibition of AGE formation (de Souza et al. 2021; Morissette et al. 2018). According to a study, puerarin injections were useful in treating DPN and increased patients’ overall effectiveness rate and nerve conduction velocity (Wu et al. 2014). According to the results, puerarin has a unique protective mechanism that reduces intracellular ROS generation, suppresses Nlrp3 inflammasome activation, and decreases subsequent caspase-1 activation, which in turn causes the release of HMGB1 (High Mobility Group Box 1) (Lian et al. 2019).
5. Anthocyanins
  (a) Pelargonidin- An anthocyanin found in many berries is known to have various properties, including antidiabetic, antimicrobial, anticancer, anti-inflammatory, and anti-obesity effects, as well as prevention of cardiovascular diseases (Khoo et al. 2017; Morais et al. 2016). Pelargonidin showed anti-hyperglycemic and protective effects on diabetic neuropathy, as shown in a paper where it reduced chemical and thermal hyperalgesia in STZ-induced diabetic rats (Mirshekar et al. 2010). Pelargonidin may reduce CCl4-induced fibrosis and TGF-β-induced HSC (hepatic stellate cell) activation by inhibiting ROS-NLRP3-IL-1β signalling through Nrf2 activation (Shi et al. 2020).
6. Flavanols
  (a) epigallocatechin-gallate (EGCG)- Epigallocatechin-3-gallate is the main active catechin in green tea and has properties including anti-oxidative, anti-inflammatory, anti-tumour, anti-diabetic, and chemopreventive properties (Farghadani and Naidu 2023; Mateen et al. 2016). Nociceptive spinal cord neurons of laminae I–III that showed signs of oxidative stress damage during diabetic neuropathy were spared by early antioxidant therapy with EGCG (Raposo et al. 2015). EGCG prevented inflammation and diabetes-induced glucose tolerance through inhibition of NLRP3 inflammasome activation (Zhang et al. 2021a, b).
  (b) Procyanidin B2 or Proanthocyanidin B2- Numerous plant parts, such as flowers, nuts, fruits, bark, and seeds, include dietary flavonoids that have a variety of antibacterial, anti-inflammatory, anti-tumour, anti-allergic, lipid-lowering, anti-obesity, and anti-diabetic qualities (Chen et al. 2022a, b). Under high-glucose-induced neurotoxicity, procyanidin B2 has been shown to improve oxidative stress, block neuronal death, promote neural regeneration, and increase the cell survival of DRG neurons (Zhang et al. 2018). Procyanidin B2 has been shown to inhibit NLRP3 inflammasome activation via suppression of the AP-1 pathway (Yang et al. 2014).
  (c) Catechins- Catechins are green tea polyphenols and are known for many properties, including antioxidant and anti-inflammatory properties, anti-cancer and anti-obesity, in various neurological and cardiovascular disorders, and anti-diabetic properties (Botten et al. 2015; Wen et al. 2022). Catechin is shown to attenuate diabetic autonomic neuropathy in streptozotocin-induced diabetic rats (‘Catechin Attenuates Diabetic Autonomic Neuropathy in Streptozotocin Induced Diabetic Rats’, 2018). Catechin has been shown to suppress IL-1β production and reduce the overactivation of NLRP3 inflammasome (Jhang et al. 2015; Jing et al. 2024).
7. Flavanonol
  (a) Silymarin- A polyphenol isolated from *Silybum marianum* (milk thistle) is shown to have many properties, including hepatoprotective, antioxidant, anti-inflammatory, anticancer, cardioprotective and anti-diabetic activities (Priya et al. 2017; Ravari et al. 2021). It has been demonstrated that giving diabetic neuropathic rats silymarin over an extended period of time improves their hyperalgesia and motor nerve conduction velocity (Baluchnejadmojarad et al. 2010) by inhibiting NLRP3 inflammasome activation and apoptosis. Silymarin has also helped in attenuating paraquat-induced cytotoxicity in macrophages by modulating the Trx/TXNIP complex by inhibiting the activation of NLRP3 inflammasome and apoptosis (Liu et al. 2018).

## Preclinical and clinical studies of flavonoids in diabetic peripheral neuropathy

Anti-inflammatory and antioxidant properties of flavonoids have made them interesting therapeutic agents for the treatment of diabetic peripheral neuropathy (DPN). Flavonoids have limitations with their structure, nature, absorption, molecular weight, toxic flavonoid-drug interactions and high first pass metabolism leading to their reduced bioavailability, therefore, by incorporating them into an effective nanocarrier system these challenges can be overcome (Caro-Ordieres et al. 2020; Dwivedi et al. 2023).

Although there is still a significant lack of well-designed research to completely prove flavonoids’ effectiveness in clinical settings, clinical trials have shown that they can lessen oxidative stress linked to diabetes and relieve neuropathic pain sensations.

The potential of natural flavonoids in the treatment of diabetes has been shown in preclinical and only a few of them in clinical investigations. In Table 2 we summarized the preclinical and clinical trial results examining the antidiabetic benefits of these bioactive substances. In therapeutic contexts, Flavones, Flavanones, Isoflavones, Flavonols, Anthocyanins, Flavanols are the primary flavonoids that have been thoroughly investigated.

**Table 2:** Preclinical and Clinical studies involving flavonoids in Diabetic Neuropathy

S. no	Flavonoid	Study Type	Dose/Treatment	Outcome	References
1	Apigenin	Preclinical	Apigenin treated doses-5, 10, and 20 mg/kg, orally and insulin (10 IU/kg) and combination of apigenin (20 mg/kg) and insulin (10 IU/kg*)* subcutaneously*,* for 4 weeks.	Combination therapy proved to have much greater effect than individual treatments in terms of hyperalgesia, allodynia and on SNCV and MNCV. Apigenin showed improvement in diabetes-induced neuropathy by reducing inflammation (TNF-α, IL-1β, IL-6) and oxidative stress through modulation of the TLR4/MyD88 signaling pathway.	Yu et al. (2023)
2	Luteolin	Preclinical	Low dose- (50 mg/kg, *i.p).,* mid dose (100 mg/kg, *i.p*.)*,* and high dose* (*200 mg/kg*, i.p.*) for 3 weeks.	Luteolin lowers blood glucose levels. It decreased the ROS and MDA levels dose dependently. It caused a significant increase in the expression of Nrf2 and HO-1 at a higher dose level than in lower dose.	Li et al. (2015)
3	Baicalein	Preclinical	30 mg kg^− 1^ d^− 1^ *i.p.*, for 4 weeks.	Baicalein improves SNCV and MNCV deficits, tactile allodynia and thermal hypoalgesia. It could not to induce intraepidermal nerve fiber regeneration despite its multi-target effect. It helped in inhibiting p38 MAPK activation and oxidative stress in diabetic neuropathy.	Stavniichuk et al. (2011); Yorek (2011)
4	Baicalin	Preclinical	Baicalin single intraperitoneal injection treated groups with doses (10 μg/kg), (20 μg/kg), and (40 μg/kg) respectively. Repeated doses of baicalin (10 μg/kg), (20 μg/kg), and (40 μg/kg) for chronic treatment were administered (i.p.) every 2 days in rats after STZ injection and lasted for 7 weeks.	A single dose of baicalin (40 µg/kg) significantly reduced pain in rats with STZ-induced DNP, and continued injections prevented the development of STZ-induced DNP in rats in a dose-dependent manner. It suppressed the expression of TRPV1 (Transient receptor potential vanilloid 1) in dorsal root ganglia in a dose-dependent manner.	Li et al. (2018)
		Clinical	Baicalin capsules given orally, 2 capsules/time, 3 times/day continuously for 8 weeks combined with α-lipoic acid.	The combination improved nerve conduction velocity, reduce oxidative stress and inflammatory injury, and does not increase adverse reactions. The levels of TNF-α, IL-6 and CRP (C-reactive protein) decreased in the combination group more than the control group with α-lipoic acid alone with saline. There was no significant difference in adverse reactions between the combination of the two drugs and the single drug, which indicated that baicalin capsules were safe and easily tolerated by patients.	Tang et al. (2021)
5	Quercetin	Preclinical	30 and 60 mg/kg/day via the intragastrical route for 6 weeks 50 mgkg^−1^day^−1^*,* intraperitoneal for 14 days, 30 mg/kg/day and 60 mg/kg/day via the intragastrical route for 6 weeks, after 8 weeks of STZ injection. 50/mg/kg and 100 mg/kg orally and naloxone (2 mg/kg *i.p.*) + quercetin (100 mg/kg) 50 mg/kg/d by gavage at 10 mL/kg continuously for 12 weeks. (10, 20 and 40 mg/kg; *p.o*.) for 8 weeks after 6 weeks of STZ induction 50 mg/kg and 100 mg/kg intragastrically once daily for 8 continued weeks.	Relieved neuropathic pain and improved nerve conduction velocity and helped downregulate TLR4, MyD88, NF-κB proteins and p65. Decreased the upregulation of the P2X_4_ receptor in the DRG and then reduced P2X_4_ receptor-mediated p38MAPK activation, relieving thermal and mechanical hyperalgesia. Improved the abnormal mitochondrial biogenesis and energy metabolism by activating AMPK and SIRT1, and promoting PGC-1α and downstream proteins, NRF1 and TFAM. Quercetin helps in attenuating thermal hyperalgesia. It is involved in the inhibition of PKC. Quercetin may reduce pain by acting on the opioid receptors, showed a protective effect by ameliorating axon and myelin damage due to oxidative stress. Reduced motor and sensory nerve conduction velocity, mechanical, mechanical allodynia and thermal hyperalgesia, superoxide dismutase, glutathione peroxidase, and oxidative-nitrosative stress decreased DNA damage and pro-inflammatory cytokines (TNF-α and IL-1β). Quercetin might help in alleviating diabetic neuropathic pain by inhibiting mTOR/p70S6K pathway.	Zhao et al. (2021; Yang et al. 2019; Zhang et al. 2021a, b; Anjaneyulu and Chopra 2003; Xie et al. 2020; Kandhare et al. 2012a, b; Wang et al. 2020)
		Clinical	Placebo or QR-333 in the ratio of 2:1, three times daily for a total of 4 weeks, Group A- Gabapentin (300 mg) once daily for three consecutive months and Group B- Gabapentin (300 mg) once daily + Quercetin mg twice daily for three consecutive months.	QR-333 helped relieve the symptoms of diabetic neuropathy and improved quality of life. It was safe and well-tolerated. The addition of Quercetin to Gabapentin showed significant improvement and had a greater effect on insulin secretion.	Valensi et al. (2005; Mohammad et al. 2022)
6	Kaempferol	Preclinical	Kaempferol (25, 50, and 100 mg/kg in DMSO, in a volume of 1 ml/100 g, initiated on the 16th day after induction of diabetes and continued for the next 21 days. 5 and 10 mg/kg of Kaempferol after 60 days of induction and continued for the next 30 days.	Kaempferol inhibits the neuroimmune activation of microglia partly by reducing the oxidative stress and production of inflammatory cytokines attenuating DNP. The treatment resulted in a decrease in blood glucose levels, an increase in the antioxidant level, and a decrease in lipid peroxidation and AGE, showing that neuropathy was relieved by affecting hyperglycemia, oxidative stress, and AGE formation.	Abo-Salem (2014; Kishore et al. 2018)
7	Myricetin	Preclinical	Starting on the 21st day of the STZ injection, intraperitoneal injections of 0.5 mg/kg/day, 1.0 mg/kg/day, and 2.0 mg/kg/day were given for two weeks.	Myricetin might have a positive effect due to Nrf2-dependent antioxidant action and ability to decrease plasma glucose.	Ma et al. (2022)
8	Morin	Preclinical	single injection of STZ (55 mg/kg, i.p. Morin administered at doses of 50 and 100 mg/kg, p.o. after 6 weeks of diabetes induction and treatment continued till 2 weeks. Single injection of STZ (65 mg/kg, *ip*), Morin administered at (15 and 30 mg/kg/day, p.o.) after 3 weeks of diabetes induction and treatment continued for 5 weeks.	By decreasing ROS-mediated IKK activation and improving Nrf2-mediated antioxidant defenses, morin assisted in reversing NF-κB-mediated neuroinflammation. Morin helped in ameliorating hyperglycemia induced thermal or mechanical hyperalgesia, lowering neuropathic pain by inhibiting oxidative stress due to its antioxidative and anti-inflammatory properties.	AlSharari et al. (2014; Bachewal et al. 2018)
9	Rutin	Preclinical	STZ injected at 65 mg/kg, ip for inducing DN, after 3 weeks of induction, Rutin was administered at low (5 mg/kg), mid (25 mg/kg) and high (50 mg/kg) intraperitoneally for 2 weeks. Other groups also received insulin and BG-12 (Nrf2 activator) and a single injection of streptozotocin (STZ, 65 mg/kg i.p.). After induction, Rutin, silymarin and rutin + silymarin administered at 100 mg/kg, 60 mg/kg and 50 mg/kg + 30 mg/kg daily orally. Single injection of streptozotocin (STZ, 55 mg/kg i.p.) and rutin administered at (100 and 200 mg/kg i.p.) and nimesulide at (5 and 10 mg/kg i.p) and given in combination with nimesulide at (100 and 5 mg/kg i.p and 200 and 10 mg/kg i.p) daily for 8 weeks.	Mid and High range dose has stronger nociceptive effect than low dose of rutin. It also helped in improving nerve conduction velocity by decreasing plasma glucose, inhibiting neuroinflammation, decreasing oxidative stress and acting as an antioxidant via Nrf2. Combination therapy of rutin with silymarin helped in ameliorating DN by their antioxidant and anti-inflammatory properties. The study shows that rutin via Nrf-2/HO-1 and NF-kB signalling pathway ameliorates the progression of the disease and the combination of rutin and nimesulide showed an additive effect by showing anti-inflammatory activity via the COX-2 inhibition.	Al-Enazi (2014; Mittal et al. 2018; Tian et al. 2016)
10	Silibinin	Preclinical	STZ injected at (55 mg kg, i.p.) and after induction, silibinin was given as treatment at 20 and 40 mg/kg orally for last 2 weeks.	Silibinin showed neuroprotective effect by activating SIRT1 and via antioxidant mechanism.	Khan et al. n.d.)
11	Taxifolin	Preclinical	Alloxan was injected at 120 mg/kg consecutively for 3 days intraperitoneally and after induction the treatment was given using taxifolin at 50 mg/kg orally once daily for 3 months.	Taxifolin helped in preventing the reduction of alloxan induced hyperglycemia -related paw pain threshold and showed antioxidant effect.	Alay et al. (2022)
12	Fisetin	Preclinical	The dose of STZ given intraperitoneally was 55 mg/kg. Following six weeks of induction, fisetin was continually given for two weeks at doses of 5 and 10 mg/kg (p.o.).	Fisetin helped in preventing oxidative stress and neuroinflammation by enhancing Nrf2 activity and decreasing NF-Κb expression.	Sandireddy et al. (2016)
13	Naringenin	Preclinical	A single dose of streptozotocin was injected at 45 mg kg^−1^. Treatment with naringenin at (25, 50, 100 mg kg^−1^, p.o.) was given in the last 4 weeks from 5 to 8th week after induction Intraperitoneal. injection of STZ was given at 60 mg/kg and treatment was started after 2 weeks using naringenin at 25 and 50 mg/kg/day for 5 weeks via the oral route. A single i.p. injection of STZ (50 mg/kg) was given to rats. Naringenin was administered at 10, 20, and 50 mg/kg orally for 8 weeks.	The study showed that the combination of naringenin with insulin modulated the oxidative stress, and inflammatory cytokine release. The study shows the anti-diabetic, anti-inflammatory and antioxidant properties of Naringenin which will help in ameliorating DN. The study shows that naringenin ameliorated diabetic neuropathy by antioxidant, hypoglycaemic, anti-inflammatory, NOS inhibitory effects and PPAR-γ (Peroxisome proliferator-activated receptor gamma) activation.	Al-Rejaie et al. (2015; Hasanein and Fazeli 2014; Singh et al. 2020)
14	Naringin	Preclinical	STZ injected at 55 mg/kg i.p and after 4 weeks chronic treatment with naringin was given at 20, 40 and 80 mg/kg for 4 weeks. Streptozotocin (STZ, 35 mg/kg BW i.p.) injected once. Naringin was administered orally for four weeks at doses of 10, 20, and 40 mg/kg after induction.	The study shows that naringenin has antiapoptotic, disease modifying property and antioxidant property by modulating endogenous biomarkers which are responsible for DN. Naringenin has shown to increase the PPAR (Peroxisome proliferator-activated receptor) and PDX-1 (pancreatic and duodenal homeobox 1) and improved beta-cell regeneration exerting its anti-diabetic effects. It also showed antioxidant, anti-inflammatory effect.	Ahmad et al. (2022; Kandhare et al. 2012a, b)
15	Genistein	Preclinical	Intraperitoneal injection of 200 mg/kg STZ was administered and genistein was administered at (3 and 6 mg/kg s.c.) for 3 weeks daily.	The study shows that genistein acts as an antioxidant, anti-inflammatory, shown to alleviate mechanical allodynia and restores the NGF content in the sciatic nerve.	Valsecchi et al. (2011)
16	Biochanin A	Preclinical	STZ was administered at a dose of 45 mg/kg i.p. Biochanin A was injected via i.p. route at doses of 1 and 5 mg/kg and given as a single injection for acute treatment at day 21 and chronic treatment was given for 7 days after the acute treatment.	The study shows that Biochanin A reversed mechanical allodynia more than mechanical hyperalgesia and has shown to be a good candidate for DN.	Chundi et al. (2016)
17	Formononetin	Preclinical	Disease was induced using High-fat diet for 15 days along with intraperitoneal administration of STZ at 35 mg/kg i.p. Treatment was given for a period of 16 weeks at 0, 20 and 40 mg/kg/day.	The study showed that formononetin reduced oxidative stress, mechanical allodynia and thermal hyperalgesia. It also upregulated SIRT1 and NGF expression in the tissues and improved the nerve conduction velocity.	Oza and Kulkarni (2020)
18	Puerarin	Clinical	Control group was administered with methylcobalamin, PGE 1, vitamin B, and nimodipine whereas the test group was administered with puerarin injection + control group medication.	The study showed that puerarin is safe clinically, it when combined with the control group drugs was more effective than the conventional therapy and helped in improving the nerve conduction velocity.	Wu et al. (2014)
19	Pelargonidin	Preclinical	A single intraperitoneal dose of 60 mg/kg of STZ was given. After one week of the disease induction, 10 mg/kg of pelargonidin was given once or every other day for eight weeks.	The study showed that pelargonidin significantly hyperalgesia compared to the untreated rats by reducing the oxidative stress.	Mirshekar et al. (2010)
20	Epigallocatechin-gallate (EGCG)	Preclinical	An intraperitoneal dose of 60 mg/kg of STZ was injected. For seven weeks, EGCG was introduced orally at 20 and 40 mg/kg every day. STZ was injected at 60 mg/kg i.p. and aqueous solution of EGCG (2 g/L) was given as treatment in the drinking water for 10 weeks after 3 days of STZ induction.	The study showed that chronic treatment with EGCG reduced the nitrite content, lipid peroxidation and improved the SOD activity and helped in improving painful diabetic neuropathy. EGCG can prevent DPN by preventing oxidative stress damage and spinal cord neuronal excitability, according to the study. It also shows that nociceptive spinal cord neurons in laminae I–III undergo oxidative stress because of diabetes.	Baluchnejadmojarad and Roghani (2012; Raposo et al. 2015)
21	Procyanidin B2/ Proanthocyanidin B2	Preclinical	Proanthocyanidin B2 was administered at 10 µg/mL on 45 mM high-glucose-cultured DRG cells.	The study shows that Proanthocyanidin B2 reversed the neurotoxicity due to high glucose levels via PI3K/Akt signalling pathway.	Zhang et al. (2018)
22	Silymarin	Preclinical	A single dose of STZ was injected at 60 mg/kg i.p. Rutin and Silymarin were administered at 100 and 60 mg/kg respectively and combination of both was administered at 50 + 30 mg/kg orally for 6 weeks after 3 weeks of induction. Single injection of STZ was administered at 60 mg/kg i.p. Treatment with Silymarin at 100 mg/kg was administered for 2 months and 200 mg/kg of silymarin was given as positive control, 1 h before conducting the formalin test.	The study shows that combination therapy showed greater protective effects than individual treatment by decreasing the antioxidant levels Silymarin ameliorated hyperalgesia, motor nerve conduction velocity and antioxidant levels.	Al-Enazi (2014; Baluchnejadmojarad et al. 2010)

## Advancement and emerging strategies

Several novel approaches can be used to enhance the absorption and oral bioavailability of flavonoids which is shown in the Fig. 1, thereby enhancing their therapeutic effects, such as using absorption enhancers, modifying the structure of compounds, Glycosylation, Prodrug approach, using various carrier complexes to improve the drug delivery system (Zhao et al. 2019). Other novel delivery approaches include, Emulsions, Co-crystals, Phytosome complexes, Co-amorphous, Solid dispersion technology, Solid lipid nanoparticles, Nanocrystals, Liposomes, Nanostructured lipid carriers, Exosomes (Yuan et al. 2024), Polymeric nanoparticles and inorganic nanoparticles (Zverev and Rykunova 2022). For the enhancement of the delivery and efficacy of treatments, nanosystems is an advanced approach. A study conducted encapsulating Apigenin in a liposome showed high entrapment efficiency (contributing to its lipophilicity) and improved stability helped in interacting of the liposome with the membrane of bacteria causing increased drug concentration intracellularly and therefore, increasing its antibacterial activity (Banerjee et al. 2015; Halevas et al. 2022). Liposomes are biocompatible and can remain in the systemic circulation for a longer period but also have less stability in the systemic circulation and high cost of production (Yuan et al. 2024). Another study used Nanostructured Lipid Carriers (NLCs) encapsulating naringenin which helped in enhancing the stability, intestinal absorption, solubility, oral bioavailability and in inhibiting the Non-alcoholic Fatty Liver Disease (Katopodi and Detsi 2021). NLCs helps in increasing the stability, solubility improve absorption by the intestine and improve the transepithelial transport but in case of partial coalescence of NLCs lipid particles start to merge causing aggregation and therefore, decreasing the stability of the system (Yuan et al. 2024). Other type of nanoparticles like Solid Lipid Nanoparticles (SLN) have also been used and are an enhanced form of lipid-based nanocarriers, they can be easily modified for a sustained and controlled release, increased intestinal absorption and have higher drug loading capacity for hydrophobic and hydrophilic drugs but have problems with their storage and flavonoids might interact with the lipids (Yuan et al. 2024; Zaheer et al. 2025). A study conducted encapsulating naringenin in SLN showed an increase in the anti-inflammatory activity (Zaheer et al. 2025). Among various nanoparticles Lipid Polymeric Nanoparticles (LPNs) are also included which have both lipid and polymer causing increased biocompatibility and enhanced stability of the molecule respectively. LPNs have high drug loading and increased entrapment efficiency (Alsulays et al. 2025). A study using LPNs helped in reducing toxicity and increasing the antitumour activity by co-delivering vinorelbine and rutin in an LPN formulation (Alsulays et al. 2025). Exosomes have been used as a novel delivery system for flavonoids and one such study utilizes epicatechin gallate encapsulated by Bovine Milk-Derived Exosomes in Parkinson’s disease which has shown to possess enhanced neuroprotective effects via anti-mitophagy and anti-apoptotic activity (Luo et al. 2021; Yuan et al. 2024). It has anti-inflammatory and anti-tumour effect but has a high purification and isolation cost. Nanocrystals and hydrogels are some other novel delivery techniques which have been used in a study where, quercetin nanocrystals loaded alginate hydrogel patch have been used for wound healing studies (Nayak et al. 2025). Hydrogels, which can hold large amounts of water and help release the drug in response to stimuli, have been used for wound healing studies, but biocompatibility and biodegradability are some of the challenges that need to be addressed. Quercetin can be made into nanocrystals with higher drug loading, which can help improve its bioavailability and aqueous solubility and using a hydrogel patch it becomes a better drug delivery system for wound healing studies (Nayak et al. 2025; Ribeiro et al. 2024; Yuan et al. 2024). These innovative methods which include a range of nanocarrier systems can provide significant advantages in enhancing the absorption, stability, and bioavailability of flavonoids, thus increasing their therapeutic potential. The successful incorporation of flavonoids into these novel nanosystems could prove essential in the treatment of conditions like diabetic neuropathy.

## Conclusion

Diabetic peripheral neuropathy (DPN) remains a significant and challenging complication of diabetes, with limited treatment options available to halt its progression. NLRP3 inflammasome is the primary cause of inflammation, which is an important player in the pathophysiology of DPN and results in oxidative stress, chronic pain, and neuronal damage. A promising approach for reducing inflammation in DPN is to target the NLRP3 inflammasome. Flavonoids, as natural polyphenols with potent anti-inflammatory and antioxidant properties, have emerged as promising therapeutic agents in this context. It has been found that altering significant pathways involved in NLRP3 inflammasome activation reduces oxidative stress and the production of pro-inflammatory cytokines like IL-1β and IL-18, preventing damage to nerves. Preclinical studies have provided compelling evidence for the efficacy of flavonoids in improving nerve function, reducing inflammation, and alleviating neuropathic pain in DPN models. Early clinical research points to flavonoids’ possible advantages for human populations. But only a small number of flavonoids have made it to market, mostly because of issues with their availability, variability and bioavailability. Novel flavonoid formulations are required to improve their medicinal efficacy and overcome this limitation. More flavonoids are also required to go through extensive clinical trials to ensure their efficacy and safety, opening the door for wider application in the treatment of disease. In conclusion, targeting NLRP3 inflammasome via flavonoid intervention may provide a novel therapeutic strategy for DPN management. Continued research using novel emerging techniques to enhance the therapeutic efficacy and clinical validation are essential to fully harness the potential of flavonoids in preventing and treating this debilitating complication of diabetes.

## Future direction

Future studies should focus on enhancing flavonoids bioavailability and therapeutic effectiveness in the treatment of diabetic peripheral neuropathy (DPN). Nanoparticles, liposomes, or micelles are examples of advanced drug delivery systems that may improve the stability, targeted administration, and prolonged release of flavonoids. Mechanistic research is necessary to determine the precise interactions between flavonoids and the NLRP3 inflammasome, which is crucial to the pathophysiology of DPN because it promotes oxidative stress, neuroinflammation, and neuronal injury. A new research avenue that offers a unique strategy to lessen the inflammatory cascade that is essential to the development of DPN is targeting NLRP3. Preclinical models must be varied to reflect different diabetes stages, comorbidities, and community variation. Furthermore, to address issues like dose standardisation and individual response variability, extensive, randomised clinical trials are required to validate the safety and effectiveness of flavonoids. New approaches to treating DPN may be made possible by investigating synergy with current treatments creating flavonoid derivatives or designed analogues and development of novel drug delivery techniques using flavonoids.

## Data Availability

All the data is publicly available, search engine that was used mainly were PubMed, Scopus, Google Scholar and Web of Science.

## Abbreviations

DN: Diabetic neuropathy
DPN: Diabetic peripheral neuropathy
NLRP3: Nucleotide-binding domain, leucine-rich-containing family, pyrin domain-containing-3
TCA: Tricyclic antidepressants
SNRIs: Serotonin and norepinephrine reuptake inhibitors
AGEs: Advanced glycation end-products
PKC: Protein kinase C
ROS: Reactive oxygen species
ASC: Apoptosis-associated speck-like protein
TLRs: Toll-like receptors
DAMPs: Damage-associated molecular patterns
PAMPs: Pathogen-associated molecular patterns
TRX: Thioredoxin-interacting protein
GSDMD: Gasdermin D
AMPK: Adenosine 5’- monophosphate-activated protein kinase
TET2: Tet methyl-cytosine dioxygenase 2
COX: Cyclooxygenase
LOX: Lipoxygenase
iNOS: Inducible nitric oxide synthase
STZ: Streptozotocin
MNCV: Motor nerve conduction velocity
SNCV: Sensory nerve conduction velocity
NF-κB: Nuclear factor-kappa B
JNK: Jun N-terminal Kinase
NLCs: Nanostructured lipid carriers
SLN: Solid lipid nanoparticles
